# Retinal glial cell removing during internal limiting membrane peeling is associated with higher-stage idiopathic epiretinal membrane

**DOI:** 10.1186/s40662-026-00514-z

**Published:** 2026-10-08

**Authors:** Denise Vogt, Christopher Schmitz, Azza Dammak, Andrea Govetto, Ricarda G. Schumann, Mario R. Romano, Melih Parlak, Armin Wolf

**Affiliations:** 1 Retina Vitreous Research Group, Department of Ophthalmology, University of Ulm, Ulm, Germany; 2 Department of Ophthalmology, University of Ulm, Ulm, Germany; 3 Department of Biomedical Sciences, Humanitas University, Pieve Emanuele, Milan, Italy; 4 Ophthalmology Department, Humanitas Gavazzeni, Bergamo, Italy; 5 Munich Eye & Vascular Medicine Center, Munich, Germany

**Keywords:** Idiopathic epiretinal membrane, Internal limiting membrane, Membrane peeling, Müller cells, Transmission electron microscopy, Vitrectomy, Vitreomacular interface, Vitreomaculopathy

## Abstract

**Background:**

Histological examination of surgically excised internal limiting membrane (ILM) specimens may provide insights into retinal tissue changes, but their relationship to retinal imaging findings remains unclear. The aim of this study was to correlate retinal imaging findings with histological evidence of retinal tissue components in surgically excised ILM specimens from eyes with idiopathic epiretinal membrane (iERM) or full-thickness macular hole (FTMH).

**Methods:**

This retrospective clinicopathological study included 42 eyes that underwent pars plana vitrectomy with membrane peeling at the Department of Ophthalmology, University Hospital Ulm, Germany, between April 2024 and May 2025: 22 eyes with iERM and 20 with FTMH. Surgically excised tissue from all eyes was examined by transmission electron microscopy (TEM), and clinical data and retinal imaging findings obtained by optical coherence tomography (OCT) were evaluated.

**Results:**

TEM confirmed the ILM in all included eyes. Intact retinal glial cells with ultrastructural features consistent with Müller cells were observed in localized areas of ILM thinning in 3 eyes with iERM but in no eyes with FTMH. Retinal cell fragments were detected in 21 eyes with iERM and 18 eyes with FTMH. Among eyes with iERM, those containing retinal glial cells had a significantly greater mean central macular thickness (CMT, 619 ± 57 µm) than those without retinal glial cells (*P* = 0.008); all 3 eyes had Stage 3 epiretinal membrane (ERM), ectopic inner foveal layers (EIFL), and central bouquet abnormalities (CBA). OCT findings did not differ according to retinal cell fragment size.

**Conclusion:**

Retinal glial cells adherent to the ILM were identified only in eyes with iERM and were associated with higher-stage ERM, often in areas of ILM thinning. By contrast, retinal cell fragments occurred in both iERM and FTMH. These findings suggest disease-specific differences at the vitreoretinal interface, with stronger and more complex retinal–ILM interactions in iERM that may influence ILM peeling.

## Background

Idiopathic epiretinal membrane (iERM) and full-thickness macular hole (FTMH) are common vitreomacular interface disorders that can cause substantial visual impairment [1]. Both involve pathological changes at the internal limiting membrane (ILM), which has a central role in their pathogenesis and surgical management [2, 3].

Histological and ultrastructural studies have shown that ILM removal during macular surgery may damage the retina [4–8]. Retinal cell fragments and intact retinal cells have been identified within or on the retinal surface of surgically excised ILM specimens from eyes with tractional maculopathies [4, 6, 9]. More recently, regions of ILM thinning have been associated with Müller cell migration through ILM pores [8]. However, the extent and nature of retinal cell involvement may differ among vitreomacular interface disorders.

In iERM, tangential traction and chronic cellular proliferation at the retinal surface may strengthen adhesion between the ILM and inner retinal layers, thereby increasing the likelihood of retinal tissue removal during ILM peeling [10–13]. By contrast, FTMH primarily results from focal anteroposterior, with a lesser contribution from less tangential traction that causes retinal dehiscence, and the interaction between the ILM and retinal tissue remains less well characterized [14–16].

Although, previous studies pioneered the ultrastructural characterization of the vitreoretinal interface using transmission electron microscopy (TEM), these foundational studies predated modern spectral-domain optical coherence tomography (SD-OCT) and current OCT-based staging systems [5, 17]. Consequently, direct clinicopathologic correlation with modern retinal imaging has historically been impossible. Our study addresses this gap by linking contemporary OCT findings with ultrastructural observations. Therefore, this study used ultrastructural analysis to compare the presence of retinal cells and cell fragments in surgically excised ILM specimens from eyes with iERM and FTMH, and correlated these findings with OCT-features.

## Methods

This retrospective clinicopathological study included 42 eyes of 42 patients: 22 eyes diagnosed with iERM and 20 with FTMH. All eyes underwent 23- or 25-gauge pars plana vitrectomy (PPV) with epiretinal membrane (ERM) and ILM peeling at the Department of Ophthalmology, University Hospital Ulm, Ulm, Germany, between April 2024 and May 2025. Surgically excised epiretinal tissue and ILM specimens from all eyes were prepared as flat mounts for light microscopy and subsequently processed into serial ultrathin sections for TEM. Clinical data and OCT findings were also analyzed.

The Ethics Committee of Ulm University approved the surgical removal and histopathological preparation and analysis of patient specimens (Approval No. 131/24). The study was conducted in accordance with the Declaration of Helsinki.

### Clinical data and retinal imaging analysis

Eyes with iERM or FTMH were eligible for clinical and retinal imaging analysis. Exclusion criteria were high myopia exceeding spherical equivalent more myopic than −6.00 D; previous intraocular surgery, except uncomplicated cataract surgery; a history of retinal tears or retinal detachment; uveitis; retinal degenerative disease; vascular abnormalities, including diabetic retinopathy; ocular trauma; or advanced cataract that impaired OCT image quality.

Clinical data were retrospectively available for all eyes at baseline and were reviewed at the 3-month and, when available, 6-month postoperative follow-up. Because of the retrospective design, baseline assessments obtained within 6 weeks before surgery were accepted, and a window of ± 6 weeks was permitted for each postoperative time point. Recorded variables included age, sex, lens status before and after surgery, and best-corrected visual acuity (BCVA). BCVA was determined by objective refraction followed by subjective refinement and was converted to the logarithm of the minimum angle of resolution (logMAR) scale for statistical analysis.

Retinal imaging was performed using SD-OCT; Spectralis, Heidelberg Engineering, Heidelberg, Germany. Macular volume scans were acquired as part of routine clinical care. Two retinal specialists (D.V. and C.S.) independently inspected retinal layer segmentation. Based on preoperative OCT findings, eyes were retrospectively classified as having iERM or FTMH according to current OCT definitions [1, 18].

On cross-sectional OCT, iERM was defined as a distinct, irregular, hyperreflective layer above the inner retinal surface and was categorized using the four-stage OCT classification described by Govetto et al. [18]. The presence or absence of ectopic inner foveal layers (EIFLs), subfoveal central bouquet abnormalities (CBA), and ellipsoid zone (EZ) disruption was recorded. EIFL denotes abnormal persistence of the inner retinal layers across the fovea, where these layers are normally absent [18]. CBA is a quantitative OCT biomarker based on the angle of the foveal pit or retinal contour and reflects the severity of retinal distortion [18]. EZ integrity was assessed by the continuity of this band, formerly termed the inner segment/outer segment line, which represents the photoreceptor inner segment–outer segment junction. The presence of microcystic macular edema (MME) and cystoid macular edema (CME) was also recorded. MME was defined as small, regular hyporeflective cystoid lesions confined to the inner nuclear layer at the perifoveolar rim. CME was defined as clusters of cyst-like spaces of varying sizes and shapes within the inner and outer nuclear layers, including the central fovea [19]. Eyes with macular pseudoholes were excluded; these were defined as ERM-associated lesions characterized by a foveal center-sparing ERM, a verticalized or steepened foveal profile, and retinal thickening.

Idiopathic FTMH was defined as disruption of all neurosensory retinal layers at the central fovea. Macular hole size was expressed as the minimum linear diameter, measured with the caliper tool at the narrowest mid-retinal point of the hole, where the edges were nearly parallel, according to established criteria [20].

Central macular thickness (CMT) was measured manually in micrometers using the manufacturer’s built-in OCT caliper tool. Measurements were obtained preoperatively and postoperatively for eyes with iERM and postoperatively for eyes with FTMH.

At each documented postoperative follow-up, CMT, EZ defects, MME, CME, and inner retinal dimpling (IRD), also termed dissociated optic nerve fiber layer), were recorded. Macular hole closure was additionally assessed in eyes with FTMH. IRD was defined as focal depressions of the inner retina on OCT B-scans.

Posterior hyaloid status was evaluated preoperatively by OCT and intraoperatively by the surgeon. On SD-OCT, posterior vitreous detachment (PVD) was classified as Stage 0, no PVD with vitreous attachment throughout the posterior pole; Stage 1, perifoveal vitreous detachment with persistent foveal and optic disc attachment; Stage 2, foveal detachment with persistent optic disc attachment; Stage 3, persistent attachment only at the optic nerve head; or Stage 4, complete detachment from both the macula and optic nerve head. Intraoperatively, PVD was classified during attempted induction as complete, partial, adherent, or strongly adherent. Complete PVD indicated spontaneous posterior hyaloid separation; partial PVD indicated incomplete separation; adherent indicated absence of spontaneous separation, with surgical induction required; and strongly adherent indicated marked resistance during induction. The surgeon also rated ILM peeling as easy to peel or difficult to remove, and this assessment was included in the analysis.

### Surgical procedure

All eyes underwent standard 23- or 25-gauge PPV with ERM and ILM peeling. When required, PVD was induced by applying suction with the vitrectomy probe around the optic nerve head. The ERM was peeled first, followed by sequential ILM peeling with end-gripping forceps over an area of at least 2 disc diameters extending to the vascular arcades. For intraoperative ILM visualization, either 0.25 mg/mL Brilliant Blue solution (Brilliant Peel; Fluoron GmbH, Neu-Ulm, Germany) or MEMBRANEBLUE-DUAL® (D.O.R.C., Zuidland, the Netherlands) was used. At the end of surgery, the vitreous cavity was filled with either balanced salt solution (BSS; Bausch and Lomb, Germany), purified air or gas (SF_6_/C_2_F_6_) as the endotamponade. Tissue specimens were maintained in BSS during transfer.

### Specimen preparation for TEM

All specimens obtained during PPV were immediately collected in Eppendorf tubes containing BSS, transported to the Ophthalmological Research Laboratory at University Hospital Ulm, and fixed in 4% glutaraldehyde.

For TEM, specimens were washed 3–4 times in for 5–10 min per wash and postfixed in 2% osmium tetroxide for 1–2 h. The specimens were then dehydrated through a graded alcohol series of 30%, 50%, 70%, and 90%, with each step lasting 2–3 min. For en bloc contrast, specimens were incubated in saturated uranyl acetate in denatured ethanol at 37 °C for at least 30 min. Dehydration was completed in 100% alcohol for 30 min, after which the specimens were incubated in propylene oxide before resin infiltration. The specimens were embedded in Epon 812 by stepwise infiltration with increasing resin concentrations and polymerized at 60 °C for 48 h. Ultrathin sections were prepared and stained with a lead-based contrast agent of uranyl acetate before TEM examination.

Ultrathin Sects. 60–70 nm thick were examined using a JEOL JEM-1400 transmission electron microscope (JEOL Ltd., Tokyo, Japan) equipped with a Veleta charge-coupled device camera (EMSIS GmbH, Münster, Germany).

The following ultrastructural features were recorded: presence or absence of the ILM, ILM thinning, retinal cell fragments, and retinal cells at the retinal side of the ILM of the ILM. Retinal cell fragments were categorized by diameter as small (< 1 μm), medium (1–2 μm), or large (> 2 μm). Cell fragments were defined as cellular debris without a visible nucleus, whereas retinal glial cells were defined as intact nucleated cells adherent to the ILM; the distinction was based on the presence of an intact cell body and visible nucleus.

### Statistical analysis

Statistical analyses were performed using IBM SPSS Statistics, version 30.0 (IBM Corp., Armonk, NY, USA). Continuous variables were summarized using the mean, standard deviation, median, range, as appropriate; categorical variables were summarized using frequencies and percentages. Inferential analyses used the Wilcoxon signed-rank test, Mann–Whitney U test, Spearman rank correlation, or Fisher exact test, as appropriate. A two-sided *P* value < 0.05 was considered statistically significant.

## Results

Table 1 summarizes the demographic and clinical characteristics as well as the OCT parameters evaluated.

**Table 1:** Demographic characteristics and optical coherence tomography (OCT) findings

Demographic characteristics and OCT findings	Idiopathic epiretinal membrane (n = 22 eyes)	Full thickness macular hole (n = 20 eyes)
Baseline		
Sex (female/male) (%)	13/9 (59%/41%)	12/8 (60%/40%)
Eye (right/left) (%)	9/13 (41%/59%)	10/10 (50%/50%)
Age (years)	70.2 ± 8.9	72.6 ± 7.2
BCVA (logMAR)	0.51 ± 0.23	0.86 ± 0.35
PVD (%) (stage 0/stage 1/stage 2/stage 3/stage 4)	9/0/4/3/6 (41%/0%/18%/14%/27%)	6/2/3/3/6 (30%/10%/15%/15%/30%)
Lens status (phakic/pseudophakic) (%)	11/11 (50%/50%)	12/8 (60%/40%)
CMT (µm)	510.7 ± 81.9	
ERM classification (stage 2/stage 3) (%)	10/12 (45%/55%)	
CBA (yes/no) (%)	9/13 (41%/59%)	
EZ defect (yes/no) (%)	0/22 (0%/100%)	
MME (yes/no) (%)	2/20 (9%/91%)	
CME (yes/no) (%)	0/22 (0%/100%)	
MME + CME (%)	3/19 (14%/86%)	
FTMH diameter (µm)	–	395.4 ± 200.9
Additional ERM in FTMH (%)	–	5/15 (25%/75%)

3-month follow-up	(n = 22 eyes)	(n = 17 eyes)
BCVA (logMAR)	0.28 ± 0.12	0.47 ± 0.34
Lens status (phakic/pseudophakic) (%)	3/19 (14%/86%)	2/15 (12%/88%)
CMT (µm)	412.1 ± 65.7	296.8 ± 38.9
EZ defect (yes/no) (%)	0/22 (0%/100%)	5/12 (29%/71%)
MME (yes/no) (%)	6/16 (27%/73%)	2/15 (12%/88%)
CME (yes/no) (%)	0/22 (0%/100%)	0/17 (0%/100%)
MME + CME (%)	0/22 (0%/100%)	1/16 (6%/94%)
IRD (yes/no) (%)	11/11 (50%/50%)	6/11 (35%/65%)
Macular hole closure (yes/no) (%)	–	17/0 (100%/0%)

6-month follow-up	(n = 16 eyes)	(n = 11 eyes)
BCVA (logMAR)	0.25 ± 0.13	0.45 ± 0.26
Lens status (phakic/pseudophakic) (%)	3/13 (19%/81%)	1/10 (9%/91%)
CMT (µm)	410.8 ± 53.1	296.6 ± 39.9
MME (yes/no) (%)	3/13 (19%/81%)	1/10 (9%/91%)
CME (yes/no) (%)	0/16 (0%/100%)	0/11 (0%/100%)
MME + CME (%)	0/16 (0%/100%)	1/10 (9%/91%)
EZ defect (yes/no) (%)	0/16 (0%/100%)	2/9 (18%/82%)
IRD (yes/no) (%)	10/6 (63%/37%)	4/7 (36%/64%)
Macular hole closure (yes/no) (%)	–	11/0 (100%/0%)

Demographical data and OCT parameters of eyes with idiopathic epiretinal membrane and FTMH at baseline and at the 3- and 6-month follow-ups

*BCVA* = best-corrected visual acuity; *CBA* = central bouquet abnormalities; *CME* = cystoid macular edema; *CMT* = central macular thickness; *ERM* = epiretinal membrane; *EZ* = ellipsoid zone defect; *FTMH* = full-thickness macular hole; *IRD* = inner retinal dimpling; *LogMAR* = logarithm of the minimum angle of resolution; *MME* = microcystic macular edema; *PVD* = posterior vitreous detachment

### Demographic, functional, and intraoperative data

At surgery, the mean age was 70.2 ± 8.9 years in the iERM group (median, 70.2 years; range, 51.3–88.6 years) and 72.6 ± 7.2 years in the FTMH group (median, 72.7 years; range, 57.1–84.3 years). Before surgery, 23 of 42 eyes (55%) were phakic, including 11 of 22 eyes (50%) with iERM and 12 of 20 eyes (60%) with FTMH. Combined PPV and cataract surgery was performed in 8 of 11 phakic eyes (73%) with iERM and 10 of 12 phakic eyes (83%) with FTMH.

In eyes with iERM, mean preoperative BCVA was 0.51 ± 0.23 logMAR and improved significantly to 0.28 ± 0.12 logMAR at the 3-month follow-up and 0.25 ± 0.13 logMAR at the 6-month follow-up (Wilcoxon signed-rank test, both *P* ≤ 0.001). In eyes with FTMH, mean baseline BCVA was 0.86 ± 0.35 logMAR, improved to 0.47 ± 0.34 logMAR at 3 months, and remained stable at 0.45 ± 0.26 logMAR at 6 months (Wilcoxon signed-rank test, *P* ≤ 0.001 and *P* = 0.003, respectively).

Table 2 summarizes the intraoperative findings. In the iERM group, complete PVD was observed in 8 eyes (36%), partial PVD in 8 eyes (36%), whereas the vitreous remained attached in 6 eyes (28%). In the FTMH group, complete PVD was observed in 5 eyes (25%), partial PVD in 7 eyes (35%), and persistent vitreous attachment in 6 eyes (30%); data were unavailable for 2 eyes. In the iERM group, ILM peeling was rated as easy in 10 eyes (45%) and difficult in 11 eyes (50%); the rating was unavailable for 1 eye (5%). In the FTMH group, ILM peeling was rated as easy in 11 eyes (55%) and difficult in 7 eyes (35%); the rating was unavailable for 2 eyes (10%).

**Table 2:** Intraoperative characteristics

Intraoperative characteristics	Idiopathic epiretinal membrane (n = 22 eyes)	Full-thickness macular hole (n = 20 eyes)
Intraoperative data		
Type of surgery (%) (PPV + peeling/PPV + peeling + phacoemulsification)	14/8 (64%/36%)	10/10 (50%/50%)
Surgical endotamponade (%) (air/SF_6_/C_2_F_6_)	21/1/0 (95%/5%/0%)	5/13/2 (25%/65%/10%)
Surgical dye (%) (Brilliant Blue/Dual Dye/n.a.)	21/1 (95%/5%)	18/1/1 (90%/5%/5%)
Triamcinolone (yes/no/n.a.) (%)	1/21 (5%/95%)	5/14/1 (25%/70%/5%)
Posterior vitreous detachment (%) (complete/partial/adherent/strongly adherent/n.a.)	8/8/4/2/0 (36%/36%/19%/9%/0%)	5/7/1/5/2 (25%/35%/5%/25%/10%)
Difficulty of ILM peeling (%) (easy to peel/difficult to remove/n.a.)	10/11/1 (45%/50%/5%)	11/7/2 (55%/35%/10%)

Ultrastructural findings	(n = 22 eyes)	(n = 20 eyes)
ILM (yes/no) (%)	22/0 (100%/0%)	20/0 (100%/0%)
Areas of well-defined ILM thinning (yes/no) (%)	7/15 (32%/68%)	7/13 (35%/65%)
Retinal cell fragments (%) (small/medium/large/no)	9/8/4/1 (41%/36%/18%/5%)	6/8/4/2 (30%/40%/20%/10%)
Intact retinal cells (yes/no) (%)	3/19 (14%/86%)	0/20 (0%/100%)

Intraoperative findings in eyes with idiopathic epiretinal membrane and full-thickness macular hole, together with ultrastructural characteristics of ILM specimens surgically excised from the same eyes

*ILM* = internal limiting membrane; *n.a.* = not available; *PPV* = pars plana vitrectomy

### Retinal imaging analysis

**Fig. 1:**
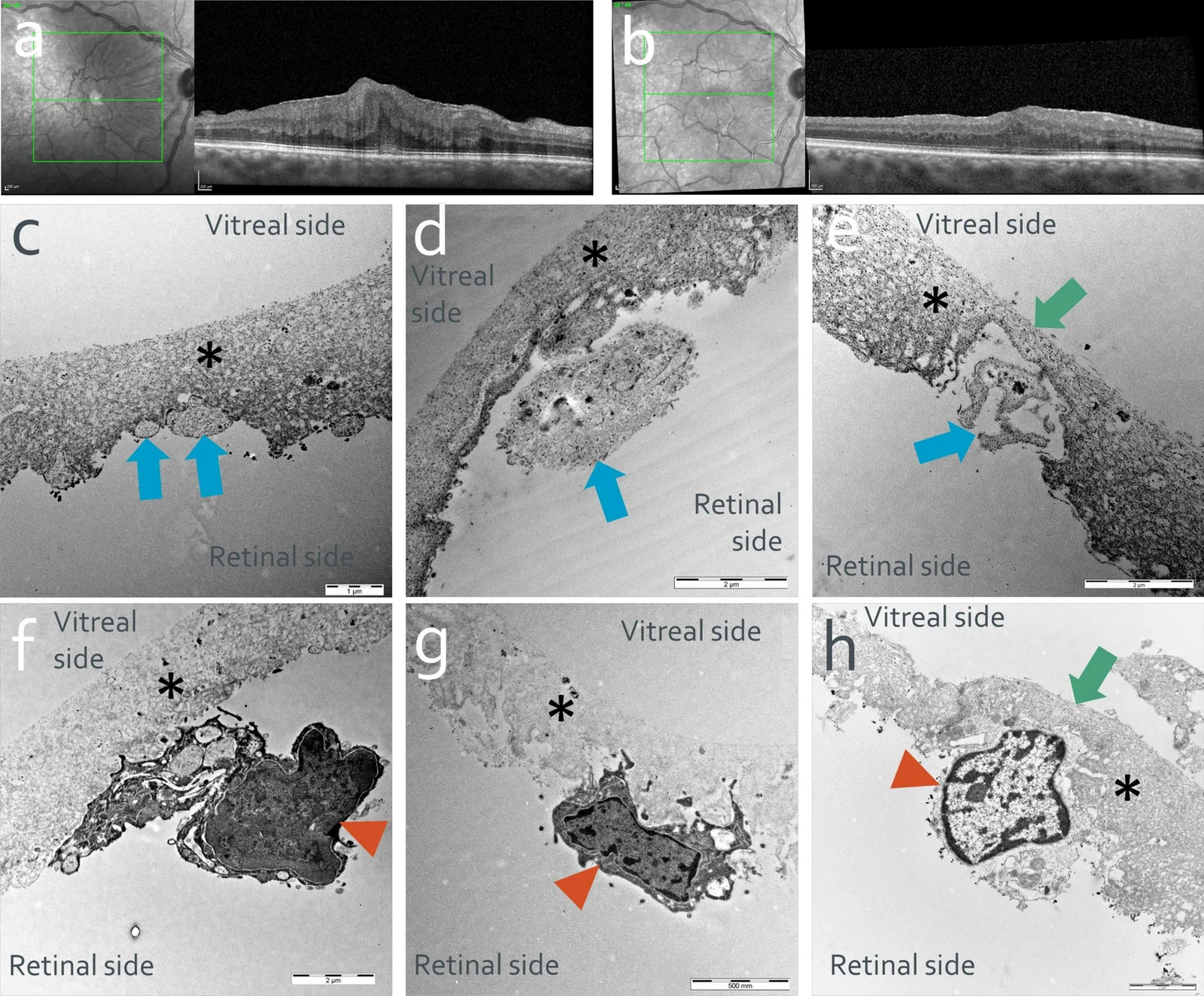
Spectral-domain optical coherence tomography image of an eye with idiopathic epiretinal membrane (iERM) (**a**) before surgery and (**b**) 6 months after surgery. **c**–**h** Transmission electron microscopy (TEM) images of eyes with iERM. TEM reveals small (**c**) and large (**d**) retinal cell fragments (blue arrows) on the retinal side of the internal limiting membrane (ILM) (asterisk). **e** These fragments are frequently observed in areas of well-defined ILM thinning (green arrow). **f**–**h** Representative TEM micrographs showing nucleated cellular profiles (red arrowheads) associated with the retinal surface of peeled ILM specimens, with cellular extensions reaching deep into the ILM and exhibiting ultrastructural characteristics consistent with Müller cells. The cellular profiles contain irregularly shaped nuclei with heterogenous chromatin and surrounding cytoplasm containing membrane-bound organelles. In (**h**), an intact nucleus with a distinct envelope is clearly visible. TEM scale bars: **c** 1000 nm; **d** 2000 nm; **e** 2000 nm; **f** 2000 nm; **g** 500 nm; **h** 2000 nm

In the iERM group, Stage 3 ERM was more common, occurring in 12 eyes (55%), whereas Stage 2 ERM occurred in 10 eyes (45%) (Fig. 1a–b). OCT-based PVD staging showed Stage 0 in 9 eyes (41%), Stage 1 in no eyes, Stage 2 in 4 eyes (18%), Stage 3 in 3 eyes (14%), and Stage 4 in 6 eyes (27%). Mean preoperative CMT was 510.7 ± 81.9 µm (median, 503.4 µm; range, 380–676 µm). EIFL were present in 12 eyes (55%), and CBA were present in 9 eyes (41%). Higher ERM stage correlated significantly with worse preoperative BCVA (Spearman ρ = 0.648, *P* = 0.001). Eyes with Stage 3 ERM also showed less postoperative improvement in BCVA than eyes with Stage 2 ERM (Mann–Whitney U test, *P* = 0.009). No eye had an EZ defect. At baseline, 3 eyes (14%) had both MME and CME, and 2 eyes (9%) had MME alone; CME alone was not observed. MME was more frequent in Stage 3 than in Stage 2 ERM (33% vs. 10%), although the difference was not statistically significant (Fisher exact test, *P* = 0.323). MME was associated with worse BCVA (Mann–Whitney U test, *P* = 0.04). At 3 months, mean CMT decreased to 412.1 ± 65.7 µm (median, 409 µm; range, 312–574 µm). MME was present in 6 eyes (27%): it persisted from baseline in 3 eyes and newly developed in 3 eyes. CME was not detected, and IRD was present in 11 eyes (50%). At 6 months, mean CMT had decreased significantly to 410.8 ± 53.1 µm (median, 407.5 µm; range, 344–554 µm; Wilcoxon signed-rank test; *P* = 0.001). MME persisted in 3 eyes (19%) that had MME at 3 months. CME was not observed, and IRD was present in 10 eyes (63%).

**Fig. 2:**
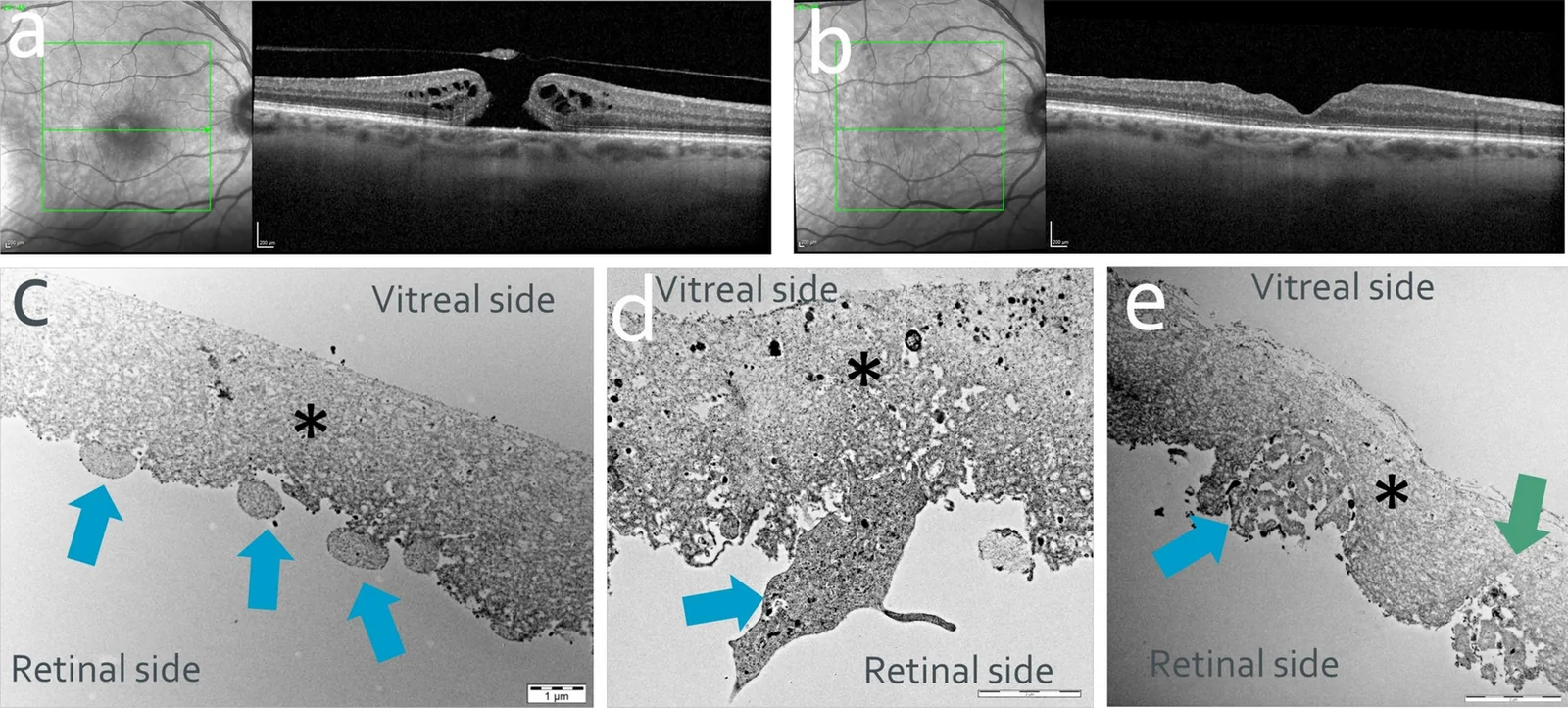
Spectral**-**domain optical coherence tomography image of an eye with full-thickness macular hole (FTMH) (**a**) before surgery and (**b**) 6 months after surgery. **c**–**e** Transmission electron microscopy images show small (**c**, blue arrows) and large (**d**, blue arrow) retinal cell fragments on the retinal side of the internal limiting membrane (ILM) (asterisk) in eyes with FTMH. **e** These fragments (blue arrow) are commonly localized to regions of well-defined ILM thinning (green arrow). Retinal glial cells, identified as nucleated cellular profiles adherent to the ILM, are not observed in FTMH specimens. TEM scale bars: **c** 1000 nm; **d** 2000 nm; **e** 2000 nm

In the FTMH group, mean baseline minimum linear diameter was 395.4 ± 200.9 µm (median, 381.5 µm; range, 117–810 µm) (Fig. 2a–b). Larger macular hole size was significantly associated with worse preoperative BCVA (Spearman ρ = 0.496, *P* = 0.026) but showed nonsignificant association with postoperative BCVA (Spearman ρ = −0.143, *P* = 0.584). OCT-based PVD staging showed Stage 0 in 6 eyes (30%), Stage 1 in 2 eyes (10%), Stage 2 in 3 eyes (15%), Stage 3 in 3 eyes (15%), and Stage 4 in 6 eyes (30%). A clearly visible ERM was present on preoperative OCT in 5 eyes (25%). At 3 months, mean CMT was 296.8 ± 38.9 µm (median, 282 µm; range, 247–397 µm). The macular hole was closed in all eyes with available follow-up data. Among eyes with available 3-month data, 1 eye (6%) had both MME and CME, 2 eyes (12%) had MME alone, 5 eyes (29%) had EZ defects, and 6 eyes (35%) had IRD. At 6 months, mean CMT was 296.6 ± 39.9 µm (median, 282 µm; range, 233–364 µm; Wilcoxon signed-rank test; *P* = 0.008). One eye (9%) had persistent MME and CME, and another eye (9%) had persistent MME alone. Two eyes (18%) had persistent EZ defects, and IRD was present in 4 eyes (36%).

### Ultrastructural analysis

TEM was used to examine specimens from all 42 included eyes. Every specimen contained a visible ILM, which appeared as a thin, acellular, lamellar structure characteristically rolled toward the vitreous side. Well-defined ILM thinning was observed in 14 of 42 specimens (33%): 7 of 22 specimens (32%) with iERM and 7 of 20 specimens (35%) with FTMH (Table 2). Thinning was confined to the retinal side of the ILM, whereas the vitreal surface remained smooth. Adjacent to the thinned regions, the ILM remained intact and retained a regular ultrastructural pattern, even at higher magnification. The thinned regions were clearly distinguishable from preparation artifacts. TEM identified no ILM breakthroughs of the kind described as ILM pores.

Retinal cell fragments were detected in 39 of 42 specimens (93%): 15 were small (36%), 16 were medium (38%), and 8 were large (19%) (Table 2). In the iERM group, fragments were observed in 21 of 22 specimens (96%): 9 were small (41%), 8 were medium (36%), and 4 were large (18%) (Fig. 1c–e). In the FTMH group, fragments were identified in 18 of 20 specimens (90%): 6 were small (30%), 8 were medium (40%), and 4 were large (20%) (Fig. 2c–e). Most fragments were round and frequently located within well-defined regions of ILM thinning. In specimens containing large fragments, these appeared to protrude from the retinal side into the thinned ILM region.

Nucleated cellular profiles on the retinal surface of peeled ILM specimens were identified in 3 of 22 iERM eyes (14%) but in no FTMH eye (Table 2). These cells extended into the ILM, and exhibited ultrastructural characteristics of glial cells. Their appearance varied with cellular state and activation. In one instance, a cellular profile exhibited a prominent, irregularly shaped electron-dense nucleus (Fig. 1f). The surrounding cytoplasm contained several membrane-bound organelles, including mitochondria (Fig. 1f). Another discrete cellular profile was characterized by an elongated, irregularly shaped nucleus with heterogenous electron density, condensed chromatin, and a thin surrounding rim of cytoplasm (Fig. 1g). Additionally, we identified an intact cellular profile containing a large oval-to-irregular nucleus with heterogeneous chromatin and prominent electron-dense regions, alongside multiple cytoplasmic organelles and surrounded by a distinct nuclear envelope (Fig. 1h).

The close association of the cellular profiles with the ILM and their processes extending into the membrane were morphologically consistent with a Müller cell–like phenotype. Retinal cells were frequently located within well-defined regions of ILM thinning, and this association was statistically significant (Fisher exact test, *P* = 0.032). No retinal nerve fiber layer fragments were identified. The cells lacked neuritic processes and did not exhibit the polarization typical of retinal neurons.

### Relationship between retinal imaging and ultrastructural findings

At baseline, eyes with iERM and retinal cells had a significantly greater mean CMT (619 ± 57 µm) than eyes without retinal cells (Mann–Whitney U test, *P* = 0.008). All three eyes had Stage 3 ERM with EIFL and CBA, although the difference in CBA was not statistically significant (Fisher exact test, *P* = 0.055). Mean BCVA in the 3 eyes with iERM and retinal cells was 0.60 ± 0.36 logMAR at baseline and did not differ significantly from that in eyes without retinal cells (Mann–Whitney U test, *P* = 0.590). Retinal cells were identified in one eye with complete vitreous detachment, one with partial vitreous detachment, and one with persistent vitreous attachment. The operating surgeon rated ILM peeling as difficult in two of the three eyes. At the 3-month follow-up, CMT remained significantly greater than in eyes without retinal cells (Mann–Whitney U test, *P* = 0.026), whereas postoperative BCVA had not deteriorated significantly (Mann–Whitney U test, *P* = 0.900). IRD was observed in one of the 3 eyes at the 3- and 6-month follow-ups. MME and/or CME were absent at baseline and at all follow-ups.

OCT findings did not differ significantly between eyes with large retinal cell fragments and those with small or medium fragments. However, among eyes with FTMH, those with large retinal cell fragments had significantly worse postoperative BCVA than those with small or medium fragments (Mann–Whitney U test, *P* = 0.023).

In eyes with iERM, ILM thinning was significantly associated with CBA (Fisher exact test, *P* < 0.001). In eyes with FTMH, there was no association between ILM thinning and larger macular hole size (Mann–Whitney U test, *P* = 0.075).

## Discussion

We identified retinal glial cells on the retinal side of the ILM only in eyes with iERM; these cells occurred in eyes with greater CMT and higher-stage ERM. The cells had ultrastructural features consistent with Müller cells and were located in regions of well-defined ILM thinning, with cellular protrusions extending into the ILM. By contrast, retinal cell fragments were present in eyes with both iERM and FTMH. These findings indicate disease-specific differences at the vitreoretinal interface. Compared with FTMH, iERM involves more pronounced and structurally complex retinal–ILM interactions, potentially reflecting disease-specific remodeling at this interface and influencing ILM peeling.

The difference between iERM and FTMH in the cellular content of surgically excised ILM specimens may reflect distinct pathophysiological mechanisms at the vitreoretinal interface. iERM is characterized by chronic tangential traction from a firmly adherent ERM–ILM complex, which may impose sustained mechanical stress on the underlying retinal surface. This prolonged traction likely promotes close adhesion between the ILM and superficial retinal cellular elements, predisposing retinal cells to displacement or removal during ILM peeling. Ultrastructural studies support this interpretation by showing that cellular material recovered from the retinal side of the ILM is associated with active epiretinal cell proliferation and remodeling at the vitreoretinal interface [4, 6]. By contrast, FTMH formation is driven primarily by focal anteroposterior traction, producing a more localized mechanical insult and comparatively less tangential stress on the surrounding retinal tissue. Moreover, because a substantial proportion of FTMH cases lack an ERM, a tightly adherent ERM–ILM complex is less likely to develop, potentially explaining the absence of retinal cells in ILM specimens from these eyes.

The detection of retinal glial cells raises the question of whether protruding Müller cells affect ERM or ILM adherence during peeling. Recent evidence indicates that retinal Müller cells can protrude into and extend through the ILM within well-defined pore regions, suggesting migration toward the vitreomacular interface [8, 21]. Müller cells are radial glial cells that span the retina; their endfeet lie adjacent to the ILM, and these cells maintain retinal homeostasis under physiological conditions [22]. On TEM, Müller cells are identified by elongated processes, intermediate filaments, dense organelle content, and specialized interactions with retinal structures, consistent with their central roles in metabolic support, structural integrity, and resistance to vitreofoveal traction [23]. Mechanical stress from vitreous traction may induce Müller cell contraction and migration, whereas retinal injury can trigger Müller cell activation, characterized by morphological changes, proliferation, and expression of inflammatory markers [23, 24]. ILM separation may therefore be more difficult in regions of Müller cell trans-ILM migration, potentially increasing the removal of retinal cellular components.

Because retinal fragments were present in both conditions, ILM peeling cannot be considered free of effect on the underlying retina. These fragments may represent passive mechanical injury rather than active cellular incorporation. However, the nature of the removed tissue differed between conditions: retinal fragments may reflect mechanical disruption common to both, whereas intact retinal cells may indicate disease-specific adhesion or integration, as observed only in iERM. Notably, Müller cell damage in tissue attached to the removed ILM did not affect visual acuity but may be associated with subtle postoperative macular dysfunction on multifocal electroretinography [9, 25]. Retinal cellular material was also associated with greater retinal thickness on OCT. This relationship requires cautious interpretation because increased retinal thickness may not cause cellular remnants to remain on the ILM; instead, it may indicate more preserved retinal tissue and thereby increase the likelihood that retinal cells or cell fragments are removed during ILM peeling.

From a surgical perspective, the stronger and more complex retinal–ILM adhesion observed in iERM than in FTMH may reflect differences in the tissue interactions encountered during ILM peeling. Traction from a contractile ERM could increase the susceptibility of the inner retina to mechanical stress during membrane removal, underscoring the need for careful surgical manipulation. In our study, intact retinal cells occurred exclusively in ILM specimens from eyes with iERM, increased CMT, and higher-stage ERM, including eyes with EIFL and CBA. These features are widely regarded as markers of chronicity and progressive tractional remodeling and reflect prolonged interaction between the ERM–ILM complex and the underlying retina [18, 26–28]. Retinal tissue involvement may therefore be associated with stronger retinal–ILM adhesion and more pronounced structural changes in higher-stage ERM. Although the clinical significance remains uncertain, this involvement could be associated with a greater likelihood of microstructural changes at the retinal surface during ILM peeling [29–31]. Whether these differences should alter surgical strategy, and whether modified ILM peeling techniques affect the extent of cellular removal at the vitreoretinal interface, remain unclear and warrant further study. Tailoring the peeling approach to disease stage and retinal morphology may help optimize surgical outcomes and preserve retinal integrity.

Our findings emphasize the importance of preoperative imaging and intraoperative assessment. Advanced OCT-based evaluation of ILM thickness and retinal–ILM interfaces may help predict adherent retinal cells and guide surgical planning; intraoperative OCT may provide additional real-time guidance to minimize retinal interaction during ILM peeling [32, 33]. Future studies should correlate these morphological findings with functional outcomes, including postoperative visual acuity and retinal sensitivity, to clarify the clinical significance of retinal cell–ILM adhesion patterns.

Several limitations should be considered. The relatively small number of surgically excised ILM specimens may limit the generalizability of the results and preclude comprehensive characterization of disease-specific differences in retinal glial cell presence and ILM morphology. Given the limited sample size and the small number of cases in specific subgroups, we did not perform multivariable analyses because they could produce overfitting and unstable estimates; analyses were therefore restricted to univariate comparisons. TEM also has inherent sampling limitations. Because TEM is labor-intensive and permits examination of only selected ultrathin sections, the entire specimen cannot be assessed comprehensively. Thus, the absence of retinal cells from the analyzed sections does not definitively exclude their presence elsewhere in the tissue, and the observed associations should be considered exploratory. Nevertheless, the consistent detection of retinal fragments in FTMH argues against a general failure of tissue detection or processing. Cytological and immunocytochemical analyses were not performed. However, no single immunocytochemical marker currently identifies Müller cells specifically. Common markers, including glial fibrillary acidic protein and vimentin, are also expressed by other cell populations. Because established ultrastructural criteria permit reliable identification of Müller cells, the study was intentionally designed to use high-resolution TEM as the primary method of cellular characterization. Accordingly, specimens were fixed in high-concentration glutaraldehyde to optimize ultrastructural preservation, which precluded subsequent immunophenotypic analyses. The incomplete availability of 6-month follow-up data is another limitation. It reflects the retrospective design and the continuation of long-term care for many patients by local ophthalmologists rather than at our tertiary referral center. In addition, the retrospective design prevented precise control of potential confounders, including disease duration, macular hole size, and traction chronicity, all of which may influence retinal involvement at the vitreoretinal interface. Larger longitudinal studies are needed to validate these findings, confirm the reported associations, and better define their clinical relevance.

## Conclusion

Clinicopathological analysis of retinal glial cells on the ILM and correlation with retinal imaging after iERM and FTMH surgery provide new insight into the pathophysiology of vitreoretinal disease. Only eyes with iERM contained retinal glial cells, with ultrastructural features consistent with Müller cells, on the retinal side of regions of ILM thinning, and their presence was associated with higher-stage ERM. By contrast, retinal cell fragments occurred within regions of ILM thinning in eyes with both iERM and FTMH; such regions have recently been associated with ILM pores [8]. Although ILM peeling may remove retinal tissue in both conditions, retinal glial cells in iERM may indicate biological differences at the vitreoretinal interface beyond a purely mechanical surgical effect. These findings elucidate disease-specific retinal–ILM interactions and may inform surgical approaches and future therapeutic strategies. Studies with larger patient cohorts and longitudinal follow-up are needed to further clarify the clinical significance of these findings.

## Data Availability

The datasets generated during and/or analyzed during the current study are available from the corresponding author on reasonable request.

## Abbreviations

BCVA: Best-corrected visual acuity
BSS: Balanced salt solution
CBA: Central bouquet abnormalities
CME: Cystoid macular edema
CMT: Central macular thickness
EIFL: Ectopic inner foveal layer
ERM: Epiretinal membrane
EZ: Ellipsoid zone
FTMH: Full-thickness macular hole
iERM: Idiopathic epiretinal membrane
ILM: Internal limiting membrane
LogMAR: Logarithm of the minimum angle of resolution
IRD: Inner retinal dimpling
MME: Microcystic macular edema
OCT: Optical coherence tomography
PPV: Pars plana vitrectomy
PVD: Posterior vitreous detachment
SD-OCT: Spectral-domain optical coherence tomography
TEM: Transmission electron microscopy
